# PROFESSIONAL QUALITY OF LIFE, LONELINESS, AND EMPATHY IN PROFESSIONAL CAREGIVERS

**DOI:** 10.1093/geroni/igz038.2363

**Published:** 2019-11-08

**Authors:** Janelle Beadle, Julie Blaskewicz Boron, Julie Masters

**Affiliations:** University of Nebraska Omaha, Lincoln, Nebraska, United States

## Abstract

Retaining professional caregivers is an ongoing challenge for home care agencies. Thus, understanding emotional factors associated with caregiver burnout is critical. While professional caregivers are at risk for burnout and loneliness, less is known about how these factors relate to empathy and professional quality of life. Professional caregivers (n=31) currently employed at two U.S. non-skilled home care agencies participated by completing several questionnaires. Surveys included: UCLA Loneliness Scale (loneliness), Interpersonal Reactivity Index (empathy), and the Professional Quality of Life Scale (compassion satisfaction, compassion fatigue, and burnout). Participants were adults (M=56.8, SD=11.5, Range=24-75 years; 90.3% female) who had obtained an Associates degree (education years: M=14.2, SD=1.7). Overall, participants reported average to high levels of loneliness (M=37.5, SD=9.8), and emotional (M=23.2, SD=3.7) and cognitive (M=20.3, SD=4.6) empathy. Caregivers generally reported positive feelings about their work (compassion satisfaction: M=42.8, SD=5.0) and low levels of burnout (M=17.1, SD=4.2). In contrast, they reported relatively high levels of compassion fatigue (M=17.7, SD=4.0). Burnout was positively associated with compassion fatigue (r=.7, p<.001), and loneliness (r=.5, p=.004), whereas a negative association was found between burnout and cognitive empathy (r=-.5, p=.008). Using a linear regression, the associations between compassion fatigue, loneliness, and cognitive empathy were examined in relation to burnout. This model was statistically significant (r2=.72; F change (3,21)=17.8, p<.001), but only compassion fatigue (p=.002) and cognitive empathy (p=.002) were significant predictors of burnout. This study highlights the importance of the contributing role of socioemotional factors to burnout experienced in a professional caregiving setting.

